# Preparation of Co-Amorphous Levofloxacin Systems for Pulmonary Application

**DOI:** 10.3390/pharmaceutics15061574

**Published:** 2023-05-23

**Authors:** Aljoscha Gabelmann, Claus-Michael Lehr, Holger Grohganz

**Affiliations:** 1 Department of Pharmacy, University of Copenhagen, Universitetsparken 2, 2100 Copenhagen, Denmark; 2 Department of Pharmacy, Saarland University, 66123 Saarbrücken, Germany; 3 Helmholtz Institute for Pharmaceutical Research Saarland (HIPS), Helmholtz Centre for Infection Research, Saarland University, Campus E81, 66123 Saarbrücken, Germany

**Keywords:** co-amorphous, spray drying, particle size, process, aerosols, dry powder inhalation, sustainability

## Abstract

Addressing antimicrobial resistance requires new approaches in various disciplines of pharmaceutical sciences. The fluoroquinolone levofloxacin (LEV) plays an important role in the therapy of lung infections. However, its effectiveness is limited by its severe side effects involving tendinopathy, muscle weakness and psychiatric disturbance. Therefore, there is a need for the development of an effective formulation of LEV with reduced systemic drug concentrations, thereby also reducing the consumption and excretion of antibiotics or metabolites. This study aimed for the development of a pulmonary-applicable LEV formulation. Co-amorphous LEV-L-arginine (ARG) particles were prepared by spray drying and characterised by scanning electron microscopy, modulated differential scanning calorimetry, X-ray powder diffraction, Fourier-transform infrared spectroscopy and next generation impactor analysis. Co-amorphous LEV-ARG salts were produced independently of varying process parameters. The use of 30% (*v*/*v*) ethanol as a solvent led to better aerodynamic properties compared to an aqueous solution. With a mass median aerodynamic diameter of just over 2 µm, a fine particle fraction of over 50% and an emitted dose of over 95%, the product was deemed suitable for a pulmonary application. The created process was robust towards the influence of temperature and feed rate, as changing these parameters did not have a significant influence on the critical quality attributes, indicating the feasibility of producing pulmonary-applicable co-amorphous particles for sustainable antibiotic therapy.

## 1. Introduction

Ensuring the pharmacologically and environmentally sustainable provision of antibiotics to address the global threat of antimicrobial resistance requires new approaches in the design and use of antibiotic drugs [1,2,3]. Excretion of drugs into the environment after administration by the patient also has to be considered due to the potentially harmful effect [4,5]; this holds especially true for antibiotics, as environmental factors can influence the spread of antibiotic resistance [6]. Therefore, reductions in antibiotic dose can be beneficial for both a reduced environmental impact from drug production and the threat of furthering antibiotic resistance. From a global perspective, pneumonia is a severe disease. Exemplified by its impact on even a well-developed nation, pneumonia is the cause of most hospital admissions for a single disease in the US, with about 1 million hospitalisations of adults, of which around 50,000 are fatal [7,8]. For children below 5 years of age, it is the world’s most deadly disease, as illustrated by about 1 million deaths in 2010 [9].

The fluoroquinolone levofloxacin (LEV) plays an important role in the therapy of lung infections, with over 3 million annual prescriptions for about 2 million patients in the US alone [10]. Unfortunately, quinolones are limited by their severe and potentially permanent adverse drug reactions involving tendinopathies such as Achilles tendon injury, muscle weakness, sleep disorder and psychiatric disturbance [11,12], apart from the classic adverse reactions for oral antibiotics such as nausea and diarrhoea. Therefore, the FDA currently recommends reserving fluoroquinolones for patients who have no alternative treatment options [13]. For cystic fibrosis patients, the standard oral and intravenous LEV dosing of 750 mg once daily is expected not to have the maximum therapeutic effect. Elevated daily dosages of ≥750 mg should hypothetically improve clinical outcomes [14]. Increasing the concentration of LEV in the body would probably increase the risk for severe adverse drug reactions. In addition, more drugs or metabolites would be provided for excretion, eventually entering the environment and contributing to the likelihood of increased antibiotic resistance [6].

To avoid the high doses of LEV necessary upon oral administration, a pulmonary application of the antibiotic could lead to a drastic decrease in drug consumption with several potential benefits. Firstly, a reduced environmental impact from production; secondly, reduced excretion and contribution to the development of antibiotic resistance; and thirdly, lower antibiotic concentrations in the body and, thereby, reduced adverse drug reactions. At the same time, pulmonary delivery may be expected to reach higher drug concentrations in the lung and the sputum, where the antibiotic effect is needed. Oral doses achieved a median concentration of 10 ± 2.5 µg/mL of levofloxacin in the epithelial lung fluid in patients suffering from idiopathic pulmonary fibrosis [15]. Considering the small volume of lung fluid of approximately 0.37 mL/kg bodyweight, those concentrations would be achieved rather easily and at a more stable level by applying only a small fraction of the typical oral dose [16].

Quinsair is currently the only inhalable LEV formulation on the market. The formulation is authorised for the treatment of *Pseudomonas aeruginosa* infections in adults with cystic fibrosis, in which it has shown a superior safety profile with incidences of adverse drug reactions at placebo levels [17]. The dosing regimen of Quinsair is 2.4 mL of a 100 mg/mL aqueous LEV solution (i.e., 240 mg, i.e., about 2/3 of the regular oral dose), and it is usually applied twice a day via a nebuliser [18]. Nebulisers have several disadvantages in comfort of use, given that they are often stationary, noisy and energy- and time-consuming in their use and cleaning. Additionally, they have a high risk of contamination, potentially leading to severe complications in cystic fibrosis patients [19,20,21]. Consequently, noncompliance among patients increases, leading to increased morbidity as well as impairment of the patient’s quality of life [22,23]. Therefore, a dry powder formulation of LEV could be an interesting alternative for the patient because of the more convenient use, which can result in better compliance. Spray drying is a commonly used technique to produce both amorphous systems and powders with particles that are suitable for inhalation [24]. A composite formulation of microparticles containing spray-dried LEV nanoparticles was investigated as an alternative approach [25]. The procedure for a nanoparticle formulation approach is often more challenging, time-consuming, less sustainable and not as easy to scale up as a simple spray drying approach. Furthermore, the drug load achieved via nanoparticle formulations is usually very low, often below 10% [25,26].

For oral delivery, LEV is a BCS class 1 drug; however, the reported solubility data for LEV varies greatly in the literature, ranging from as low as 1.44 mg/mL [27] to as high as 300 mg/mL, with some reports indicating solubility levels in the range of 20 mg/mL [28], probably due to its pH dependency. However, in any case, an increased solubility will increase the driving force for cellular uptake and considering the very small volume of lung fluid in which the drug must be dissolved, high solubility and a fast dissolution rate are considered an advantage for pulmonary delivery. A common strategy to increase the apparent solubility of BCS class 2 drugs is the transformation of a crystalline drug to its amorphous counterpart. Drugs in their amorphous form often have increased apparent solubility and dissolution rate, with potential supersaturation, compared with their crystalline state, but are thermodynamically unstable. Various formulation approaches are available to address this inherent stability, frequently based on amorphous solid dispersions, such as polymer-based amorphous solid dispersions, mesoporous systems or co-amorphous systems. Co-amorphous systems have shown their potential to overcome these stability issues while maintaining the improved physicochemical properties of the amorphous form [29]. Amino acids are the most common excipients used as co-formers, as they are generally regarded as safe and can provide a wide range of different functional groups to enhance the formation of a co-amorphous system [30]. Spray drying as a production technique has been investigated thoroughly for the production of co-amorphous systems; thereby, spray drying has the double effect of being suitable for both the intended pulmonary application and the intended drug system. The potential of the combination of the co-amorphous platform with pulmonary antibiotic delivery has recently been realised.

The antibiotic ceftazidime was spray-dried together with several amino acids. The authors reported a positive effect of the various amino acids on aerosol performance and reduced chemical degradation. Homogeneous amorphous systems with one glass transition temperature (T_g_) were reported; however, the T_g_ of the co-amorphous systems was not increased notably compared with the T_g_ of the pure ceftazidime. The FTIR analysis also indicated no strong interactions in the investigated co-amorphous systems, except for the co-amorphous system with tryptophan, where interactions of the aromatic ring with the drug were postulated [31]. Another approach combined the antibiotic ciprofloxacin with quercetin in order to combine the antibiotic with antibiofilm properties. Moreover, in this study, which addressed many aspects of the co-amorphous system, improved stability and improved aerosol performance were observed. The ratio between the drug and the co-former was reported to impact the solid state of the system. The T_g_s of the pure amorphous compounds were not investigated, but compared to the literature data; however, all co-amorphous systems showed extremely high T_g_s, also above the expected T_g_s based on the applied Fox equation. Paradoxically, the T_g_ evolution across the investigated ratios did not indicate the formation of robust interactions. Both FTIR and NMR studies were conducted, and the occurrence of interactions was discussed but not conclusively proven [32].

Spray drying is easily up-scalable for an industrial purpose, and the characteristics of the produced particles can be influenced in several ways, indicating it to be a promising production technique for the project under investigation [33]. Several approaches have shown that it is possible to produce co-amorphous systems with L-arginine (ARG) as a co-former by spray drying [34,35]. ARG was chosen as a co-former as salt formation between the basic guanidine group of ARG and the carboxylic acid group of LEV was expected. Salt formation further increases the stability of the co-amorphous system and enhances the solubility of the compounds [36]. The current approach would result in a high drug loading of 67% (*w*/*w*). So far, very few dry powder formulations of LEV have been investigated. Lawlor et al. [37] earlier investigated two LEV formulations, including leucine and other excipients. They achieved MMADs of around 5 µm and T_g_s of 58 °C and 79 °C, respectively. Other aerodynamic powder properties such as emitted dose and fine particle fraction were not reported. Furthermore, Cayli et al. developed dry powders of combinations of fluoroquinolones and mucolytic agents specifically for cystic fibrosis patients and focused on the influence of different agents on particle properties and particle penetration through mucus [38]. LEV-loaded polymer microspheres based on chitosan and their optimisation were investigated by Gaspar et al. with a factorial approach. The produced particles exhibited a MMAD greater than 5 µm, thus mainly targeting the upper lung [39].

It was thus the aim of the project to investigate whether the formation of co-amorphous inhalable particles of LEV and ARG with increased apparent solubility by spray drying was a feasible approach. Furthermore, the study aimed to understand and engineer the spray drying process to gain the ability to fine-tailor the desired aerodynamic properties of the resulting LEV dry powder formulations based on application-targeted lung areas. The ultimate goal of this project was to provide a stepping stone for an improved antibiotics treatment with reduced consumption and excretion of antibiotics.

## 2. Materials and Methods

### 2.1. Materials

LEV (Mw 361.4 g/mol) and ARG (Mw 174.2 g/mol) of reagent grade were purchased from Sigma-Aldrich (St. Louis, MO, USA) and used as received. Ethanol absolute 99.7% was purchased from VWR Chemicals (Rosny-sous-Bois-cedex, France).

### 2.2. Spray Drying (SD)

Initially, equimolar amounts of LEV and ARG (resulting in a mass ratio of 67.4% LEV and 32.6% ARG) were dissolved in 100 mL of water. In the later course of the study, LEV and ARG were dissolved in 100 mL of a 30% ethanol/water mixture (*v*/*v*). The solid concentration was 10 mg/mL. SD was performed using a Büchi B-290 spray dryer (Büchi Labortechnik AG, Flawil, Switzerland), which was equipped with an inert loop B-295 (Büchi Labortechnik AG) when ethanol was used as a solvent. A 0.7 mm two-fluid nozzle was used. To investigate the influence of the process parameters on the outcome, samples were produced at varying inlet temperatures and feed rates in a design of experiments (DoE) set-ups. The drying air flow and the atomising air flow rate were kept constant at 40 m^3^/h (nitrogen) and 667 l/h, respectively.

### 2.3. X-ray Powder Diffraction (XRPD)

XRPD was performed using a X’Pert PANalytical PRO X-ray diffractometer (PANalytical, Almelo, The Netherlands) using Cu Kα radiation (λ = 1.54187 Å) and acceleration voltage and current of 45 kV and 40 mA, respectively. The samples were scanned in reflectance mode between 5° 2θ and 35° 2θ with a scan speed of 0.067° 2θ/s and a step size of 0.026° 2θ. Data were collected and analysed using the software X’Pert Data Collector (PANalytical, Almelo, The Netherlands).

### 2.4. Fourier-Transform Infrared Spectroscopy (FTIR)

Infrared spectroscopic measurements of LEV-ARG mixtures were performed on a MB3000 Fourier transform infrared spectrometer (ABB, Saint-Laurent, QC, Canada) equipped with a MIRacle ATR sampling accessory with a ZnSe crystal (PIKE Technologies, Madison, WI, USA). The infrared spectra were collected with Horizon MB software (version 3.2.5.2, ABB, Saint-Laurent, QC, Canada). Spectra were collected from 600 to 4000 cm^−1^ and calculated as a mean of 64 spectra with a resolution of 16 cm^−1^. Reference spectra of the surrounding atmosphere were obtained as a mean of 64 spectra with a resolution of 16 cm^−1^.

### 2.5. Scanning Electron Microscopy (SEM)

SEM images were taken with a TM 3030 Tabletop Microscope (Hitachi, Tokyo, Japan) after coating the samples with gold with a Cressington Sputter Coater 108 auto (TESCAN GmbH, Dortmund, Germany).

### 2.6. Thermogravimetric Analysis (TGA)

TGA analysis was performed on a Discovery TGA (TA Instruments, New Castle, DE, USA). The platinum pans were loaded with approximately 10 mg of sample and heated up from room temperature to 200 °C with a heating rate of 2 °C/min. Weight–temperature diagrams were analysed using TRIOS software (version 5.1.1, TA Instruments, New Castle, DE, USA).

### 2.7. Modulated Temperature Differential Scanning Calorimetry (mDSC)

Modulated DSC was performed with a Q2000 DSC (TA Instruments, New Castle, DE, USA). Approximately 5 mg of sample was weighed into an aluminium pan and sealed with a pinhole lid. Two different DSC methods were applied: In a heat-only run, the samples were heated to 190 °C at a heating rate of 2 °C/min with a modulation amplitude of 0.25 °C and a period of 60 s. To reduce the impact of moisture, a heat–cool–heat run was also performed as follows: Samples were first heated from room temperature to 100 °C at a heating rate of 5 °C/min and kept isothermal for 5 min to remove moisture from the powder. The samples were then chilled down to zero degrees, the data storage was turned on and the samples were heated to 190 °C at a heating rate of 2 °C/min with a modulation amplitude of 0.25 °C and a period of 60 s. Thermograms were analysed with TRIOS software (version 5.1.1, TA Instruments, New Castle, DE, USA). The glass transition temperature (T_g_) was analysed as the midpoint of the event.

### 2.8. Next Generation Impactor (NGI)

The aerodynamic properties of the samples were investigated with a Next Generation Impactor (MSP Corporation, Shoreview, MN, USA). Size 3 Vcaps Plus dry powder inhaler (DPI) capsules (Lonza, Basel, Switzerland) were loaded until the larger part was filled with approximately 40–50 mg of sample (accurately weighed) and activated in an Aerolizer DPI. For NGI experiments, flow rates between 30 and 100 L/min are considered suitable. The airflow was set to the maximum flow rate of the available pump at 52 L/min and activated for 2.4 s. The powder was recovered by washing the stages three times with 4 mL of water. The DPI, throat section and preseparator were washed 3 times with varying amounts of water. The analysis of the results was carried out according to the NGI manual:

The cutoff diameters were calculated with Equation (1):

(1) $$D_{50}=D_{50}(at\;60\frac{L}{min}){\left(\frac{60}{airflow\left[\frac{L}{min}\right]}\right)}^{x}$$

Values for the D_50_ at 60 L/min and for x were taken from the NGI manual (Table 1):

The capsule was weighed before and after actuation. The emitted dose (ED) was calculated as the weight difference between these two measurements. The fine particle fraction (FPF) was set to be the portion of the particles with an aerodynamic diameter of 5 µm and below. To calculate the mass median aerodynamic diameter (MMAD), the cumulative mass was plotted vs. the cutoff diameters of stages 1–7. In the resulting diagram, the measurement points right above and below 50% of the cumulative mass were interpolated. The resulting straight equation was solved for y = 50, as the MMAD occurs where the cumulative per cent is 50% [40].

### 2.9. Quantitative Analysis

For the quantification of LEV on each NGI stage, the solutions obtained from the NGI were diluted, and the amount of levofloxacin was determined with an Evolution 300 UV-Visible-Spectrophotometer (ThermoFisher Scientific, Waltham, MA, USA) at a wavelength of 292 nm. The limit of detection and limit of quantification with this method were determined to be around 0.6 µg/mL and 1.8 µg/mL, respectively. Due to the variation in the literature data, the solubility of crystalline LEV was analysed at three relevant pH values of 6.6, 6.8 and 7.2 by measuring the supernatant of saturated solutions with the above method. The apparent solubility of the amorphous material was investigated by dissolving LEV-ARG masses corresponding to defined degrees of supersaturation in 2M HEPES buffer. The supersaturated solutions were observed until precipitation occurred.

### 2.10. Design of Experiments

The set-up and evaluation of the statistical design of experiments were conducted with the software MODDE 13 (Sartorius, Göttingen, Germany). As a starting point, a full factorial design with two factors (inlet temperature and freed rate) on two levels was chosen. As the setting with low inlet temperature and high feed rate (−1;+1) resulted in an overwetted product, this corner point was replaced by two points with one intermediate setting in each (−1;0) and (0;+1).

## 3. Results and Discussion

### 3.1. Spray Drying from Aqueous Solution

**Figure 1:**
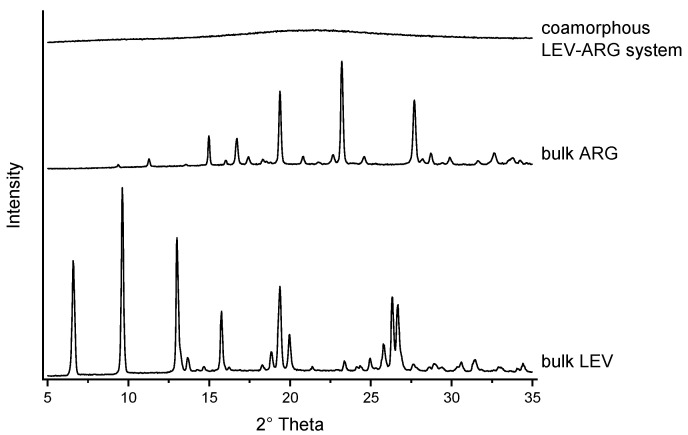
XRPD analysis of spray-dried formulation from aqueous solution compared with LEV and ARG starting material.

The initial experiment was carried out based on an aqueous solution of LEV and ARG due to the high solubility of the compounds in the solvent. Based on a trial run, an inlet temperature of 130 °C and a feed rate of 4 mL/min were applied. This resulted in the formation of a bright yellow powder, corresponding to the yellow colour of the LEV starting material. XRPD confirmed that the sample was in an amorphous form (Figure 1).

**Figure 2:**
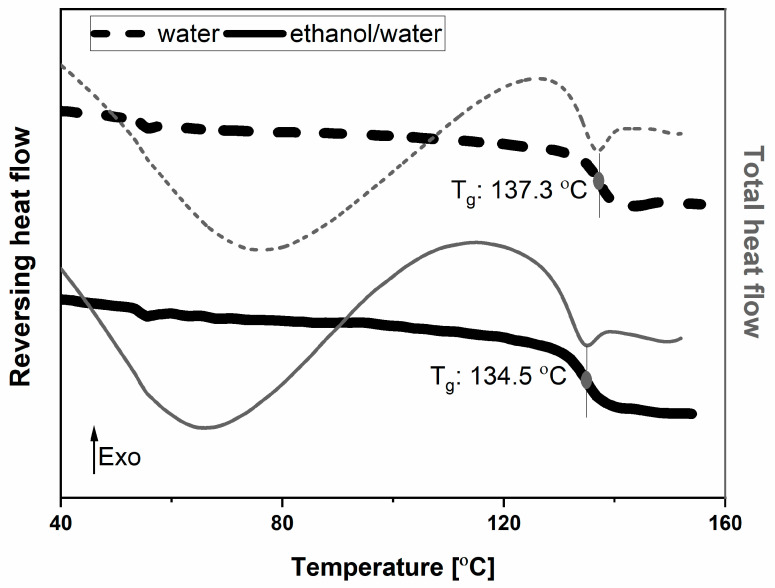
mDSC of spray-dried material from water and from ethanol/water mixture (DoE centre point). Bold, black line resembles reversing heat flow, thin grey line resembles total heat flow.

Following this confirmation, it was of interest whether the obtained amorphous system consisted of a single phase. For this means, mDSC was applied. Analysis of the sample with both modulated DSC protocols showed a single T_g_ at around 137 °C, confirming that a homogeneous co-amorphous system of LEV-ARG was obtained (Figure 2, Supplementary Figure S1). A relatively high T_g_ is usually an indicator of good stability of the co-amorphous system [41]. Considering the T_g_s of nonsalt amorphous LEV systems, which have been reported between 47 °C and 79 °C [37,42], respectively, and the T_g_ of 55 °C of ARG [43], the observed T_g_ for the co-amorphous system was found to be much higher than could be expected according to the Gordon–Taylor equation. The Gordon–Taylor equation is used to describe the T_g_ of binary mixtures. It is based on the supposition that no interactions arise between the components of the system. Interactions between the two components generally result in positive deviations of the experimentally determined T_g_ from the predicted T_g_ [43]. 

**Figure 3:**
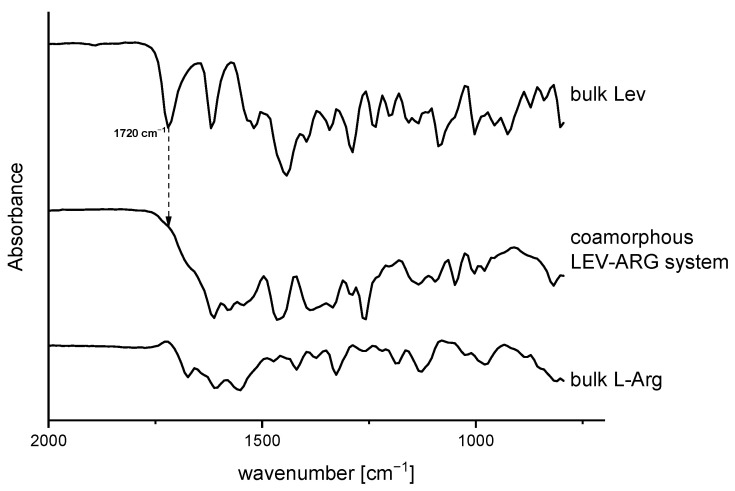
FTIR spectra of spray-dried co-amorphous LEV-ARG-system (**middle**) compared with LEV starting material (**top**) and ARG starting material (**bottom**).

For the present case, this behaviour is likely due to the formation of a co-amorphous salt. Considering the pKa values of the two components, 6.25 (pKa_1_ LEV) [27] and 13.2 (pKa_3_ ARG) [44], salt formation between the carboxylic group of LEV and the guanidinium group of ARG is likely to happen. This assumed salt formation was confirmed by FTIR, based on the disappearance of the signal of the unbound –COOH bond of LEV at 1720 cm^−1^ (Figure 3).

**Figure 4:**
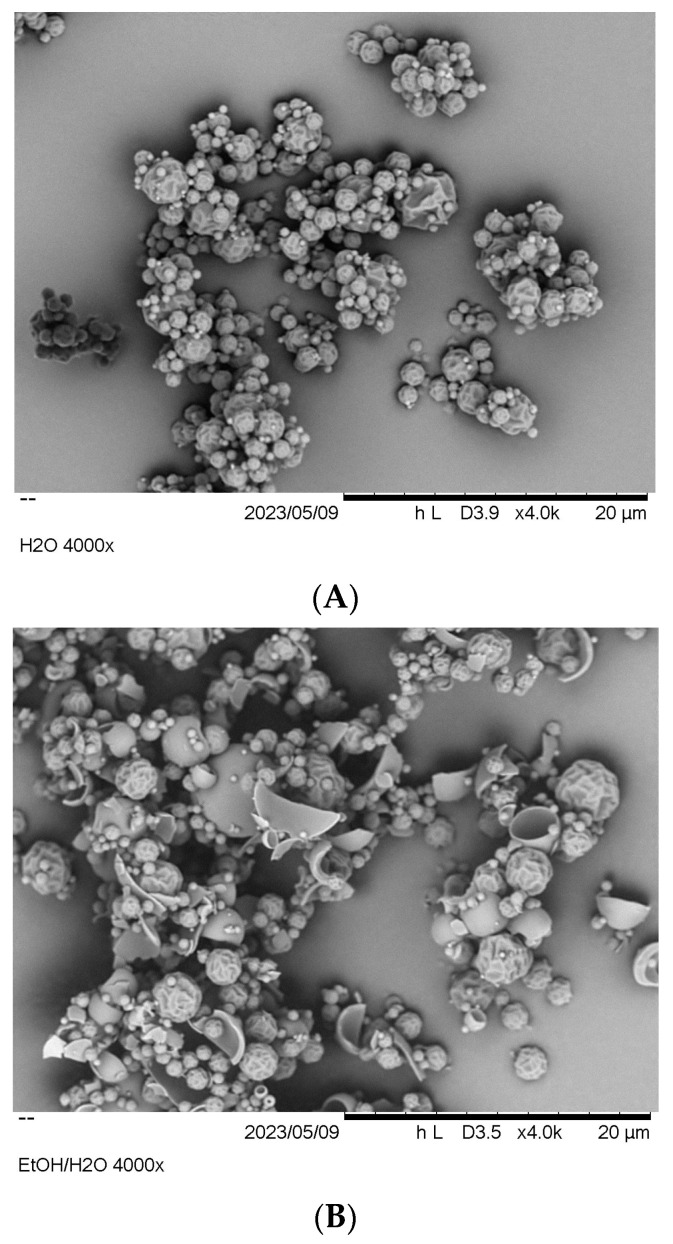
SEM images of spray-dried LEV-ARG particles obtained from (**A**) water as solvent and (**B**) ethanol/water mixture at 3 k magnification.

The SEM images (Figure 4) of the sample showed round but wrinkled particles in the size range of between 0.5 and 5 µm, with the occasional appearance of a hole in the shell.

**Figure 5:**
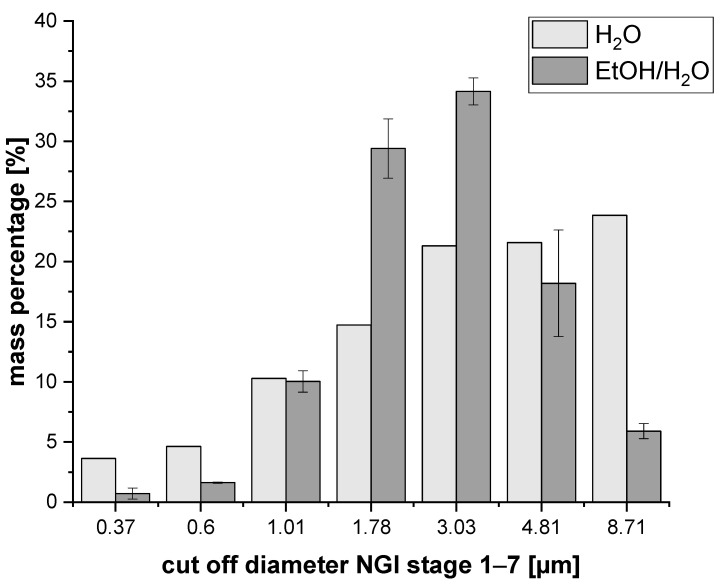
Comparison of the size distributions of particles spray-dried from H_2_O vs. 30% EtOH/H_2_O. Displayed are the percentages of the total powder recovered from the NGI.

Investigation of the aerodynamic parameters with the NGI showed a MMAD in the desired range between 0.5 and 5 µm (see Figure 5 for the detailed distribution). Particles with an observed MMAD of 2.76 µm can be expected to enter the lung but are not quite yet in the best range to majorly reach the deep lung tissues. For that goal, a MMAD between 0.5 and 2 µm is targeted. The emitted dose was acceptable, with a value of 79%, but it had the potential for improvement. However, a large proportion of the powder was found in the preseparator and throat section, probably due to a high amount of residual moisture of around 9% (see Supplementary Table S1 and Figure S2). As a consequence, the fine particle fraction was relatively low at only 23%. Thus, alternative approaches were worthy of being investigated. It can be concluded here that spray drying from water as a solvent showed a promising result, with possible improvements regarding particle characteristics. Considering the aim of the manuscript, the formation of a homogenous co-amorphous LEV-ARG system was shown to be feasible. With regard to the pulmonary application, formulation and process optimisation were thus worth considering.

### 3.2. Spray Drying from EtOH/H_2_O Mixture

**Figure 6:**
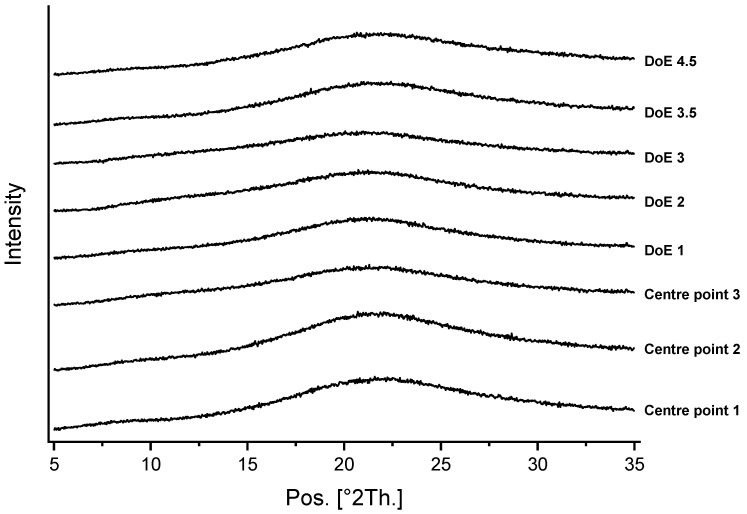
XRPD diffractograms showing the formation of amorphous systems for all investigated formulations included in the DoE.

In order to obtain particles with a larger FPF, a solvent with a higher evaporation speed was applied. The solvent was changed to 30% ethanol/water (*v*/*v*) whilst the spray drying parameters were kept constant. To investigate process repeatability, three repetitions were performed with the previously described process settings. XRPD and FTIR confirmed that, again, a homogeneous co-amorphous LEV-ARG salt was obtained for all three repetitions (see Figure 6 for XRPD).

The solubility of crystalline LEV was found to vary only slightly in the range from 28.5 mg/mL via 22.5 mg/mL to 18.6 mg/mL at pH values of 6.6, 6.8 and 7.2, respectively. The intermediate value of 22.5 mg/mL was used for the evaluation of the achieved supersaturation of the co-amorphous LEV-ARG system. The duration of the concentration-dependent achieved supersaturation is given in Table 2.

It can be seen that a substantial increase in apparent solubility was observed. As can be expected, the duration of maintained supersaturation decreased with an increasing degree of supersaturation. Nevertheless, a remarkable duration of supersaturation of around 1 day was observed for a degree of supersaturation of four, whilst even a very high degree of supersaturation of eight could be maintained for 20 min.

The DSC graph revealed a similar T_g_s compared with the initial experiment of around 135 °C (Figure 2). The deviations between the centre points were minor and might be attributed to slight variations in the remaining moisture content.

The SEM image (Figure 4B) showed a slightly different appearance of the particles. The particles still had about the same size and a wrinkled surface. However, hollow and shattered particles were observed to a larger extent. This can be explained as follows: due to the lower solubility of LEV and ARG in ethanol compared to water, the particle formation starts earlier during spray drying, forming a shell and thus causing more solvent to be trapped inside the particles. This solvent finally evaporates at later drying stages. The built-up pressure leads to a fast release of gas, thereby creating a hole in the shell or shattering the particle.

The aerodynamic properties of the particles spray-dried from the ethanol/water mixture also differed from the original approach. In between the three repetitions, the MMADs differed a little, with 1.94 µm, 2.09 µm and 2.22 µm. Nevertheless, a clear shift towards lower MMADs was recognisable, approaching the desired range of 0.5–2 µm. As the particle size did not seem to change drastically in the visual evaluation of the pictures, the shift towards the lower aerodynamic diameters is assumed to be caused by the lower density of the particles. A substantial increase in the emitted doses to over 95% was found for all three samples, pointing towards a superior production process. Despite some variation in the three samples, the FPF showed a strong increase to 52–59% compared with 23% for the aqueous approach. This increase is in good correlation with the recovered mass percentage in the NGI stages of the total recovered mass (Supplementary Table S1).

In addition to the above, a narrower size distribution was also found. Figure 5 shows the mass percentages vs. the cutoff diameters of the different NGI stages. The size distribution became smaller for the ethanol approach, and the main proportions of the particles were located at later stages, namely stage 3 (3.03 µm) and 4 (1.78 µm) of the NGI. Summarising the above, the exchange of an aqueous solution with an EtOH/H_2_O mixture improved the particle properties with regard to the pulmonary application while still providing a homogeneous co-amorphous system of LEV and ARG.

### 3.3. Investigation of Process Parameters

To further investigate the robustness and modification possibilities of the created process, a design of experiments (DoE) approach was set up. A full factorial design at two levels was chosen as the starting design. The two process parameters, ‘inlet temperature’ and ‘feed rate’, were chosen to be of the highest importance for product quality based on prior studies [45]. The prior setting was used as the centre point for the DoE study. Three of the four new runs also resulted in the formation of comparable spray-dried powders. In contrast, when a high feed rate was combined with a low inlet temperature, the resulting product was wet and ultimately destroyed by solvent condensing in the sample collector. To compensate for the missing point, the design was changed to include two additional points, being placed at an intermediate setting combined with an extreme setting, yielding an accepted condition number for the model of 2.33. The settings for the process parameters are shown in Table 3.

At all the updated process parameter settings, comparable solid-state properties were obtained. The obtained powders were characterised as homogeneous co-amorphous LEV-ARG according to the XRPD (Figure 6). The T_g_s of 137–139 °C did not differ from the T_g_s found at the centre point.

In addition, the particulate properties did not show a recognisable difference in the appearance of the particles in the SEM images either. All conditions resulted in particles of approximately the same size, with some hollow and shattered particles in between.

**Figure 7:**
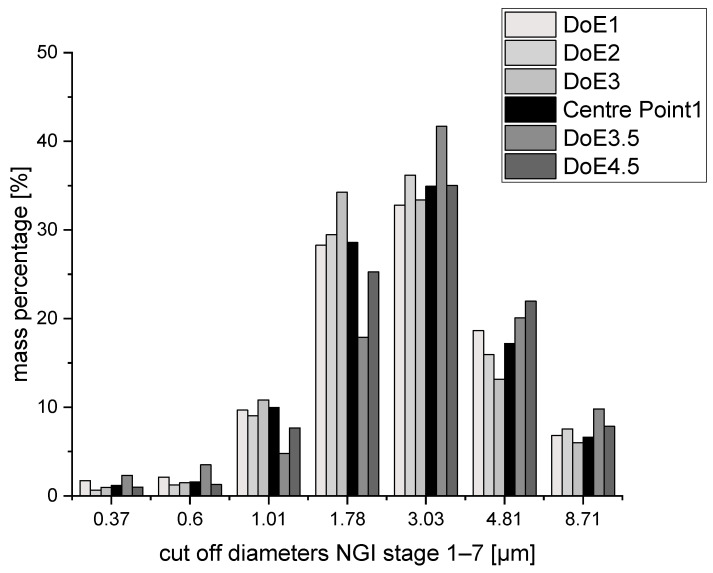
Size distributions of all EtOH/H_2_O approaches.

NGI data showed that there was some fluctuation between the aerodynamic characteristics resulting from the various DoE settings (Table 3). In order to obtain an applicable model, significant model terms are to yield in a model with a high goodness of fit (R^2^ preferably above 0.9) and goodness of prediction (Q^2^ in close relation to R^2^). However, statistical analysis with MODDE showed that there was no significant influence of any of the investigated factors—inlet temperature and feed rate—on the process outcome in the investigated range. The emitted dose remained over 95% for all the samples, and the FPF varied from 47 to 55%. The MMADs were in the range from 1.87 µm (DoE 3) to 2.43 µm (DoE3.5), which is only a −0.07 µm difference to the lowest and +0.21 µm difference to the highest MMAD of the three centre points, whilst the variation in the centre points themselves was ±0.15 µm. The size distributions also gave a similar picture for all parameter settings (Figure 7). The detailed masses obtained at each stage for every individual sample are displayed in Table S1.

It can thus be concluded from the statistical experimental approach that the process parameters did not have a significant influence on the investigated quality attributes. For all cases, particles with a MMAD around 2 µm, emitted doses above 95% and FPF generally above 50% were obtained, thus indicating a process that is robust towards changes in the input settings.

## 4. Conclusions

Spray drying enabled the production of a co-amorphous LEV-ARG salt with a high T_g_ of around 137 °C. Compared with the crystalline drug, a substantial increase in apparent solubility was found and maintained for prolonged periods. The addition of ethanol as a solvent to the spray-dried solution resulted in an improvement in the intended application. The majority of the spray-dried powder consisted of special particles with a diameter below 5 µm. The aerodynamic properties showed a MMAD close to 2 µm, a high emitted dose and a FPF of usually above 50%. The powders, thus, have suitable aerodynamic properties for a potential pulmonary delivery. The process was found to be robust towards the influence of temperature and feed rate in the investigated design space and is thereby suitable for creating the desired product. In conclusion, spray-dried co-amorphous systems might be an approach to reducing the use of antibiotics and the risk of antibiotic resistance via their local application of lower doses.

## Figures and Tables

**Table 1:** Parameters for calculating D_50_ values for stages of the NGI between 30 L/min and 100 L/min according to NGI manual.

Stage	D_50_ at 60 L/min [µm]	x
1	8.06	0.54
2	4.46	0.52
3	2.82	0.50
4	1.66	0.47
5	0.94	0.53
6	0.55	0.60
7	0.34	0.67

**Table 2:** Achieved duration before precipitation occurred for various degrees of supersaturation of co-amorphous LEV-ARG in comparison to plain LEV (22.5 mg/mL).

Corresponding Concentration of LEV (mg/mL)	Degree of Supersaturation	Time until Precipitation Was Observed (Hours)
90	4	23
112	5	8
135	6	2
180	8	0.33

**Table 3:** Overview of process parameters and aerodynamic properties obtained from the DoE set-up. The initial run was spray-dried from water, while all other samples were spray-dried from 30%EtOH-water mixtures.

Sample	Inlet Temp. [°C]	Feed Rate [mL/min]	MMAD [µm]	ED [%]	FPF [%]
Initial run	130	4	2.76	79	23
Centre points	130	4	1.94, 2.09, 2.22	98, 96, 96	53, 59, 52
DoE 1	145	6	2.09	95	53
DoE 2	145	2	2.11	98	55
DoE 3	115	2	1.87	97	56
DoE 4	115	6	/	/	/
DoE 3.5	115	4	2.43	98	47
DoE 4.5	130	6	2.31	99	55

## Data Availability

Data available on request.

## Supplementary Materials

The following supporting information can be downloaded at: https://www.mdpi.com/article/10.3390/pharmaceutics15061574/s1, Figure S1: DSC thermogram originating from the second heating in the heat-cool-heat cycle; Figure S2: Thermogravimetric analysis of spray-dried material from H_2_O and from EtOH/H_2_O mixture (DoE center point); Table S1: Mass recovery from different NGI stages.
