# Supplementary material for: Preparation of Co-Amorphous Levofloxacin Systems for Pulmonary Application
Source: Pharmaceutics. 2023 May 23;15(6):1574. doi: 10.3390/pharmaceutics15061574 (PMC10302593; doi:10.3390/pharmaceutics15061574)
Supplement: Supplementary file 1 [file pharmaceutics-15-01574-s001.zip › pharmaceutics-2369208-supplementary.pdf]

Supplementary information

Preparation of co-amorphous levofloxacin systems for pulmo-nary application

Aljoscha Gabelmann, Claus-Michael Lehr, Holger Grohganz

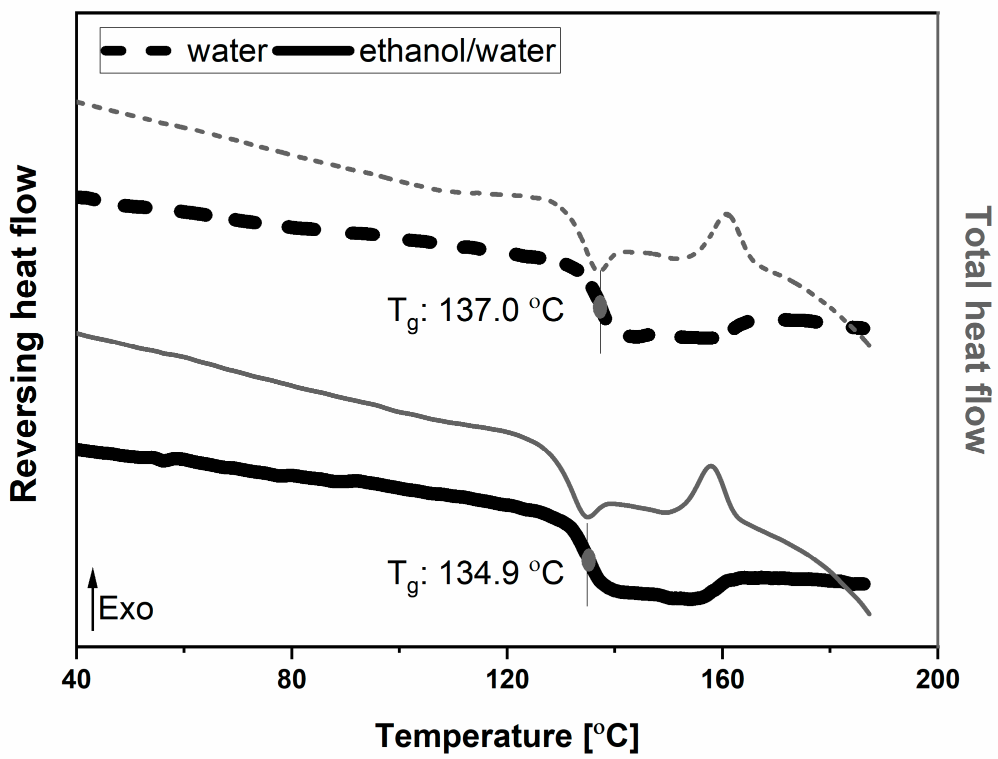

Figure S1. DSC thermogram originating from the second heating in the heat-cool-heat cycle

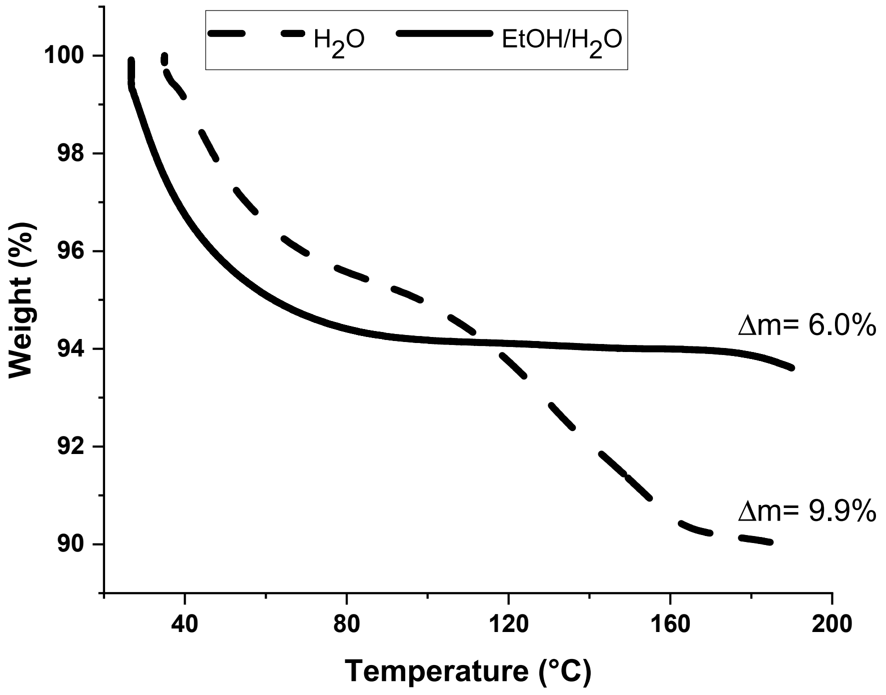

Figure S2. Thermogravimetric analysis of spray-dried material from H_2O and from $EtOH/H_2O$ mixture (DoE center point)

Table S1. Mass recovery from different NGI stages

	H_2O	Center 1	Center 2	Center 3	DoE 1	DoE 2	DoE 3	DoE 3.5	DoE 4.5
Presep + throat	15757	11838	14627	16279	15596	8310	13683	16246	19808
1	1536	1344	1005	1124	1369	694	1201	1713	2265
2	1390	3489	4149	2937	3742	1685	2633	3506	6335
3	1372	7092	5915	7086	6588	4867	6683	7286	10103
4	948	5805	4939	6580	5678	5187	6856	3123	7293
5	662	2023	1652	2240	1942	1662	2165	834	2211
6	298	319	298	336	423	316	294	613	367
7	233	237	43	151	345	246	191	402	283
MOC	65	123	13	75	156	128	116	141	134
